# Jian-Pi-Yi-Shen Regulates EPO and Iron Recycling Protein Expressions in Anemic Rats with Chronic Kidney Disease: Accumulation of Hypoxia Inducible Factor-2*α* via ERK Signaling

**DOI:** 10.1155/2020/8894257

**Published:** 2020-10-30

**Authors:** Fochang Wang, Huimin Yu, Shiying Huang, Lin Zheng, Ping Zheng, Shangbin Zhang, Shunmin Li, Jianping Chen

**Affiliations:** ^1^The Fourth Clinical Medical College of Guangzhou University of Chinese Medicine, Shenzhen 518033, China; ^2^Shenzhen Key Laboratory of Hospital Chinese Medicine Preparation, Shenzhen Traditional Chinese Medicine Hospital, Shenzhen 518033, China; ^3^School of Medicine, Shenzhen University, Shenzhen 518060, China

## Abstract

Jian-Pi-Yi-Shen (JPYS), the traditional Chinese medicine (TCM) decoction, has been commonly used to treat chronic kidney disease (CKD) and its complications such as anemia. JPYS has been previously found to induce erythropoietin (EPO) production in HEK293T cells and CKD rats. However, the mechanism of JPYS in treating anemia of CKD rats has remained largely unknown. Here, we further extend our effort to investigate the translational control of hypoxia inducible factor- (HIF-) *α* protein via ERK signaling and the effect on iron recycling-related protein expression by JPYS, thus revealing the mechanism of JPYS in correcting anemia in CKD. Experimental CKD rats with anemia were induced by 5/6 nephrectomy. Rats were administrated orally with high dose (6.0 g/kg/d) and low dose (1.5 g/kg/d) of JPYS for 90 days. Serum hepcidin level was determined to evaluate iron homeostasis. The protein expressions of HIF-2*α*, erythropoietin (EPO), ferritin, and ferroportin (FPN) and the phosphorylation level of extracellular signal-regulated kinase 1/2 (ERK1/2) were detected by Western blot. The results showed that JPYS treatment significantly ameliorated kidney function by reducing increased levels of blood urea nitrogen (BUN), serum creatinine (Scr), and urine protein (UPRO). Periodic acid-Schiff (PAS) and Masson staining observation showed that the renal pathological damage was restored in JPYS-treated CKD rats. In parallel, JPYS markedly improved CKD anemia through upregulation of red blood cell (RBC), hemoglobin (HGB), and hematocrit (HCT). JPYS stimulated EPO and HIF-2*α* protein expressions in both the kidney and liver of CKD rats. Furthermore, JPYS induced the phosphorylation of ERK1/2 protein. In addition, JPYS regulated protein expression of ferritin and FPN in both the liver and spleen of CKD rats and the serum level of hepcidin. In conclusion, JPYS induces the expression of EPO through ERK-mediated HIF-2*α* protein accumulation and regulates systemic iron recycling, supporting its role in promoting erythropoiesis and improvement of anemia in CKD.

## 1. Introduction

Anemia is the most common complication of chronic kidney disease (CKD) [1, 2], and CKD anemia is associated with a poor quality of life and elevated cardiovascular mortality [3]. Inadequate production of erythropoietin (EPO) and iron metabolism disorders are considered as the two main causes that contribute to anemia in advanced CKD [4, 5]. On the one hand, the hypoxia inducible factor (HIF) as a transcription factor regulates 500–1000 gene expressions including EPO [6]. Accumulation of HIF-*α* protein plays crucial roles in increasing transcriptional activity of HIF-regulated genes, resulting in induction of EPO expression. Stabilization and accumulation of HIF-*α* protein is required to be translocated to the nucleus, where HIF-*α* nuclear accumulation and transcriptional activity were found to be regulated by ERK1/2 [7]. Notably, the kidneys of patients with CKD retain the ability to produce EPO [5]. Besides, liver-derived EPO synthesis could contribute to the circulating EPO level as a deficient kidney-derived EPO synthesis due to the loss of kidney function [8]. On the other hand, humans need iron every day to synthesize new red blood cells. The absorption of iron in the diet is rather limited, and most of the iron is provided by macrophages recycling from senescent red blood cells [9]. However, functional iron deficiency due to inefficient utilization of iron stores can lead to the development of anemia in CKD patients [10]. At present, the treatment of CKD patients who experienced anemia with erythropoiesis-stimulating agents (ESAs) has greatly improved their quality of life in clinical practice. This treatment, however, has been associated with adverse effects, such as increased cardiovascular events, promotion of progression or worse outcomes in several cancers, and low or no response to ESAs in some patients [11]. In addition, for some CKD patients requiring dialysis, intravenous iron supplementation is needed for those who suffer from iron deficiency, yet its safety concerns also remain [12]. Therefore, it is still needed to find a more effective and safe approach for the treatment of CKD anemia.

Traditional Chinese medicine (TCM) plays a unique role in the treatment of many diseases, and a growing body of evidence has shown that TCM is increasingly being used for preventing CKD and anemia [13–15]. Jian-Pi-Yi-Shen (JPYS), a Chinese herbal formula, is composed of eight medicinal herbs, that is, Astragali Radix, Atractylodis Macrocephalae Rhizoma, Amomi Fructus Rotundus, Dioscoreae Rhizoma, Cistanches Herba, Rhei radix et Rhizoma, Salviae miltiorrhizae Radix et Rhizoma, and Glycyrrhizae radix et Rhizoma Praeparata cum Melle. JPYS has been prescribed for treating CKD for over 20 years in clinics. Previous cellular and animal experiments have revealed that JPYS stimulated the transcriptional expression of EPO in HEK293T cells and improved the anemia symptoms by increasing the serum EPO level and HIF-*α* protein in 5/6 nephrectomized rats [16, 17]. These findings present that activation of HIF signaling occurs in JPYS-induced EPO expression in CKD anemia.

However, the specific mechanism involved remains to be further investigated. Besides, regulation of JPYS in the utility of available iron stores in response to iron deficiency in anemia and CKD needs to be elucidated. Based on the importance of EPO and iron homeostasis in the occurrence and development of anemia in CKD, the roles of JPYS on translational control of HIF-*α* protein via ERK signaling and iron recycling could be hypothesized. We therefore aim to further investigate the mechanism of JPYS in the CKD anemia rat model. Specifically, the outcomes of EPO expression and anemia symptoms are determined. And then, the translational control of HIF-*α* protein via ERK signaling will be first revealed in JPYS-treated CKD rats. In addition, the effect on iron recycling-related protein expression by JPYS will be investigated.

## 2. Materials and Methods

### 2.1. Drugs

JPYS was obtained from the Pharmaceutical Department of Shenzhen Traditional Chinese Medicine Hospital (Lot no. 180813). JPYS consists of eight herbs, and the mixed proportion of the respective herb is Astragali Radix (30 g, roots of *Astragalus membranaceus* (Fisch.) Bge. var. *mongholicus* (Bge.) Hsiao), Atractylodis Macrocephalae Rhizoma (10 g, rhizomes of *Atractylodes macrocephala* koidz.), Dioscoreae Rhizoma (30 g, rhizomes of *Dioscorea opposita* Thunb.), Cistanches Herba (10 g, fleshy stems with scaly leaves of *Cistanche deserticola* Y.C. Ma), Amomi Fructus Rotundus (10 g, fruits of *Amomum kravanh* Pierre ex Gagnep.), Salviae miltiorrhizae Radix et Rhizoma (15 g, roots and rhizomes of *Salvia miltiorrhiza* Bunge.), Rhei radix et Rhizoma (10 g, roots and rhizomes of *Rheum palmatum* L.), and Glycyrrhizae radix et Rhizoma Praeparata cum Melle (6 g, roots and rhizomes of *Glycyrrhiza uralensis* Fisch.). JPYS was prepared as previously described [18]. Briefly, Amomi Fructus Rotundus, Atractylodis Macrocephalae Rhizoma, Glycyrrhizae radix et Rhizoma Praeparata cum Melle, Rhei radix et Rhizoma, and 1/3 of the Dioscoreae Rhizoma were crushed together into a superfine blend powder. The remaining three herbs and 2/3 of the Dioscoreae Rhizoma were extracted with boiling water twice for 1 h. After centrifugation, the supernatant was concentrated under reduced pressure and which was further mixed with the prepared powder to form the JPYS sample. The yield of the extract was∼30.8%. HPLC-MS/MS chromatograms of the JPYS extract are shown in Supplementary Figure 1, which guarantee the quality of JPYS. The sample was freshly prepared and dissolved in ddH_2_O before use.

### 2.2. Animals

10-week-old Sprague-Dawley (SD) rats (24 males and 24 females, 200 ± 20 g) were purchased from Guangdong Medical Laboratory Animal Center (Foshan, China). They were maintained in a specific pathogen-free (SPF) animal facility with free access to food and water. The rats were housed under a constant temperature (23 ± 2°C) and humidity (55 ± 15%) with a 12-hour light/12-hour dark cycle. This animal study was approved by the Institutional Animal Care Use Committee of Guangzhou University of Chinese Medicine, and institutional guidelines for the care and use of laboratory animals were followed.

### 2.3. Establishment of the CKD Anemia Model in Rats

Rats were conducted by a 5/6 nephrectomy to induce CKD anemia [19]. More specifically, the abdominal cavity was opened through an incision on the right back. After the renal pedicle was clamped by a vein clip, the upper and lower poles of the right kidney was removed with electrocautery, and only left one-third of the right kidney. A second operation was performed 2 weeks later to remove the left kidney. The sham operation group took the same steps to open the abdominal cavity and restore the kidney after exposure to avoid pulling the kidney. All rats were intraperitoneally injected under anesthesia with 10% chloral hydrate saline (3 ml/kg body weight). The rats were randomly assigned to 4 groups in average: (1) sham group (Sham), (2) renal anemia group (RA), (3) low dose JPYS-treated group (LJPYS, 1.5 g/kg/d), and (4) high dose JPYS-treated group (HJPYS, 6.0 g/kg/d). After 90 days of operation, rats in the treatment groups were administered through gastric gavages, while rats in sham and RA groups were treated with an equal volume of ddH_2_O for totally 90 days. After the last administration, urine for 24 h was collected; then, the rats were anesthetized and euthanized for sampling. Blood samples including whole blood and serum were obtained from the abdominal aorta. Kidneys, livers, and spleens were collected and observed for pathological analysis and Western blotting analysis.

### 2.4. Biochemical Parameters Examination

Whole blood levels of white blood cell (WBC), red blood cell (RBC), hemoglobin (HGB), and hematocrit (HCT) were detected by the automatic hematology analyzer (SIEMENS 2120i, Erlangen, Germany). Urine protein (UPRO), serum urea nitrogen (BUN), creatinine (Scr), calcium (Ca), and phosphorus (P) were measured by the detection kits according to the manufacturer's specifications (Nanjing Jiancheng Institute of Biotechnology, Nanjing, China). Serum hepcidin was determined by enzyme-linked immunosorbent assay (ELISA) according to the manufacturer's instruction (Shanghai Enzyme-Linked Biotechnology, Shanghai, China).

### 2.5. Histopathological Examination

After fixation with 10% neutral formalin solution for 48 h, the rat kidney tissues were routinely dehydrated, paraffin-embedded, and sectioned. The sections were then subjected to PAS staining and Masson staining, respectively. Pathological examination and quantitative analysis were performed under an optical microscope (Zeiss, Oberkochen, Germany). The scoring method of tubular atrophy in PAS staining was described previously [17]. In brief, a four-point scale was evaluated: 0, normal tubules; 1, rare single atrophic tubule; 2, several clusters of atrophic tubules; and 3, massive atrophy. Image J software (NIH, Bethesda, USA) was used to evaluate renal interstitial fibrosis in Masson staining. In each section, 10 microscopic fields were randomly selected for observation at 200x microscope to observe glomerular changes and measure the extent of tubular atrophy and the interstitial fibrosis area.

### 2.6. Western Blot Analysis

Tissue lysis was conducted in RIPA lysis buffer containing protease inhibitors (MCE, USA) and phosphatase inhibitors (Thermo, USA). Total protein extracts were determined by the BCA Protein Assay kit (Beyotime Biotechnology, China), added 4× loading buffer (Bio-Rad, USA) and heated for 10 min at 100°C. Equal amounts of protein extracts were separated through 10% sodium dodecyl sulfonate-polyacrylamide gel (SDS-PAGE) and then transferred onto nitrocellulose (NC) membranes (Millipore, USA). After the NC membranes were blocked at room temperature under 5% nonfat milk for 1 h, the membranes were washed by TBST for 10 min and incubated with primary antibodies at 4°C overnight. The membranes were washed with TBST for 3 times and then incubated with the secondary antibodies (Life Technologies, USA) at room temperature for 1 h and washed repeatedly. Finally, the expression of proteins was determined by the Tanon imaging system (Tanon, Shanghai, China). The primary antibodies included HIF-2*α* (Abcam, USA), EPO (Santa Cruz, USA), ERK1/2 (CST, USA), *p*-ERK1/2 (CST, USA), Ferritin (Abcam, USA), FPN (Abcam, USA), PHD1 (Abcam, USA), PHD2 (Novus, USA), PHD3 (Novus, USA), *β*-actin (Abcam, USA), and GAPDH (Proteintech, USA).

### 2.7. Statistical Analysis

SPSS 22.0 software was used to analyze and process the data. The data of each group was expressed as mean ± standard deviation (SD). Statistical significance among groups was performed by one-way ANOVA and post hoc analysis with the least significant difference (LSD) test or Dunnett's T3 test. The value of *P* less than 0.05 was considered statistically significant.

## 3. Results

### 3.1. JPYS Attenuates Kidney Injury

**Figure 1:**
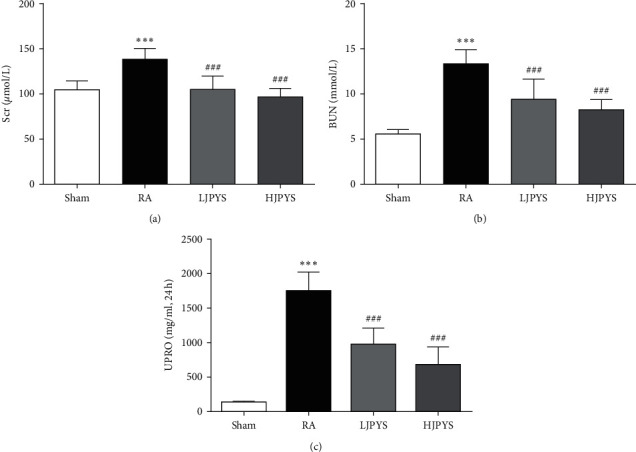
Effects of JPYS on kidney function in rats. The levels of (a) Scr, (b) BUN, and (c) UPRO from different groups. The results were presented as the means ± standard deviations (*n* = 6; ^∗∗∗^*P* < 0.001 compared with the sham group; ^##^*P* < 0.01, ^###^*P* < 0.001 compared with the RA group). JPYS, Jian-Pi-Yi-Shen formula; Scr, serum creatinine; BUN, blood urea nitrogen; UPRO: urine protein; RA, renal anemia; LJPYS, low dose Jian-Pi-Yi-Shen (1.5 g/kg/d); HJPYS, high dose Jian-Pi-Yi-Shen (6.0 g/kg/d).

To examine the effect of JPYS on kidney damage, the biochemical parameters related to renal function was determined. After 90 days of gavage, the levels of Scr, BUN, and UPRO were significantly increased in the RA group compared with the sham group. And the increased Scr level was remarkably decreased in JPYS-treated groups, with a similar observed for BUN and UPRO levels (Figures 1(a)–1(c)). CKD is commonly associated with serum calcium (Ca) and phosphorus (P) disorders. We further found that the levels of Ca and P were significantly increased in the RA group compared with the sham group, and the levels of both were significantly reduced in JPYS-treated groups compared with the RA group (Supplementary Figure 2).

**Figure 2:**
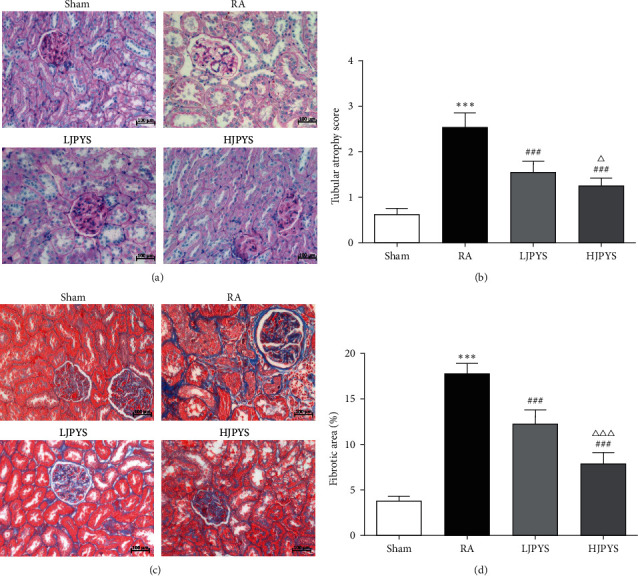
Effects of JPYS on histopathological examination in rats. (a) Changes of renal atrophy among different experimental groups in pas staining (×200, scale bar = 100 *μ*m). (b) Renal tubular atrophy score. (c) Changes of renal interstitial fibrosis among different experimental groups in Masson staining (×200, scale bar = 100 *μ*m). (d) Quantification of renal interstitial fibrosis. The results were presented as the means ± standard deviations (*n* = 6; ^∗∗∗^*P* < 0.001 compared with the sham group; ^###^*P* < 0.001 compared with the RA group; ^Δ^*P* < 0.05, ^ΔΔΔ^*P* < 0.001 compared with the LJPYS group). JPYS, Jian-Pi-Yi-Shen formula; PAS, periodic acid Schiff; RA, renal anemia; LJPYS, low dose Jian-Pi-Yi-Shen (1.5 g/kg/d); HJPYS, high dose Jian-Pi-Yi-Shen (6.0 g/kg/d).

Furthermore, the renal pathological changes of rats in various groups were detected by PAS and Masson staining. PAS staining outcomes showed that the RA group had severe glomerular hypertrophy and tubular atrophy compared with the sham group, while the JPYS treatment groups dose-dependently ameliorated the glomerular hypertrophy and tubular atrophy in the kidney of rats (Figures 2(a) and 2(b)). Massive EPO-producing cells were distributed in the renal interstitium, and interstitial fibrosis destroyed the production of EPO [20]. In the Masson staining, fibrotic area was robustly enlarged in the RA group compared with the sham group. Compared with the RA group, kidney fibrosis was significantly attenuated in JPYS treatment groups in concentration-dependent manner (Figures 2(c) and 2(d)).

### 3.2. Effects of JPYS on Hematological Parameters

**Figure 3:**
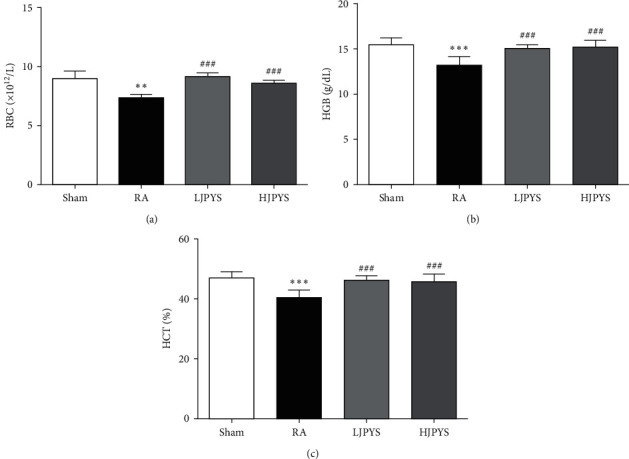
Effects of JPYS on the hematological parameters. The levels of (a) RBC, (b) HGB, and (c) HCT in blood from different groups. The results were presented as the means ± standard deviations (*n* = 6; ^∗∗^*P* < 0.01, ^∗∗∗^*P* < 0.001 compared with the sham group; ^###^*P* < 0.001 compared with the RA group). JPYS, Jian-Pi-Yi-Shen formula; RBC, red blood cell; HGB: hemoglobin; HCT: hematocrit; RA, renal anemia; LJPYS, low dose Jian-Pi-Yi-Shen (1.5 g/kg/d); HJPYS, high dose Jian-Pi-Yi-Shen (6.0 g/kg/d).

To analyze the effect of JPYS on anemia symptoms, the hematological parameters were investigated. As shown in Figure 3, levels of RBC, HGB, and HCT were significantly decreased in the RA group compared with the sham group, confirming that anemia was successfully induced in 5/6 nephrectomized rats. After treatment with the JPYS extract, the declined levels of RBC, HGB, and HCT restored to near normal. These results showed that JPYS could improve the anemia symptoms of 5/6 nephrectomized rats.

### 3.3. JPYS Increases the Proteins Expression of HIF-2*α* and EPO

**Figure 4:**
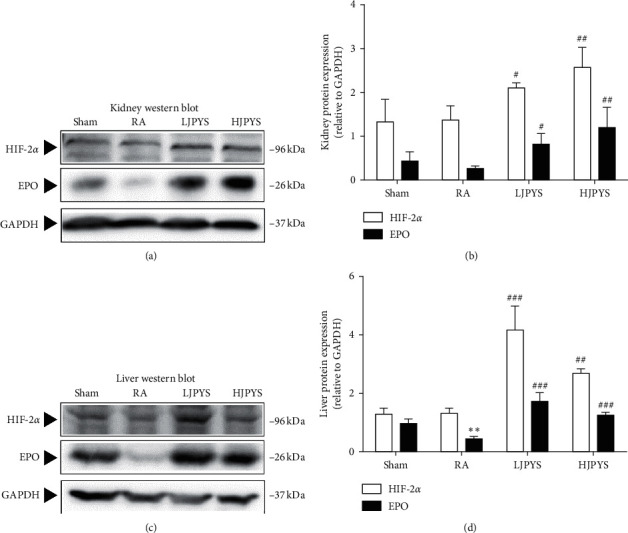
Effects of JPYS on the proteins expression of HIF-2*α* and EPO in rats. (a) Representative Western blot images of HIF-2*α* and EPO in the kidney. (b) Kidney proteins expression of HIF-2*α* and EPO relative to GAPDH. (c) Representative Western blot images of HIF-2*α* and EPO in the liver. (d) Liver proteins expression of HIF-2*α* and EPO relative to GAPDH. The results were presented as the means ± standard deviations (*n* = 3; ^∗∗^*P* < 0.01 compared with the sham group; ^#^*P* < 0.05, ^##^*P* < 0.01 and ^###^*P* < 0.001 compared with the RA group). JPYS, Jian-Pi-Yi-Shen formula; HIF-2*α*, hypoxia inducible factor-2*α*; EPO, erythropoietin; GAPDH, glyceraldehyde-3-phosphate dehydrogenase; RA, renal anemia; LJPYS, low dose Jian-Pi-Yi-Shen (1.5 g/kg/d); HJPYS, high dose Jian-Pi-Yi-Shen (6.0 g/kg/d).

To explore the potential mechanism of JPYS, the protein expressions of HIF-2*α* and EPO in the kidney and liver were detected by Western blot. As indicated in Figure 4, the expression of HIF-2*α* in CKD rats was not statistically different from that of sham rats, both in the kidney and liver. The kidney protein level of EPO was slightly declined in CKD rats compared with sham rats, but this difference in the liver was statistically significant. Both HIF-2*α* and EPO levels in CKD rats were significantly increased after JPYS administration. These results showed that JPYS increased the level of EPO protein in the kidney and liver tissues, which might relate to the accumulation of HIF-2*α* signaling.

**Figure 5:**
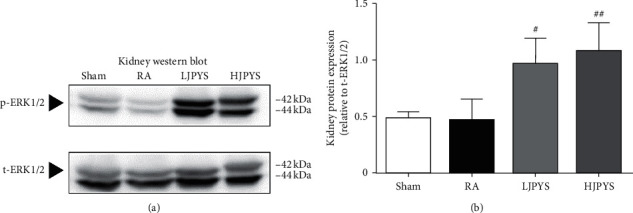
Effects of JPYS on p-ERK1/2 protein expression in rats. (a) Representative Western blot images of p-ERK1/2 in the kidney.(b) Kidney protein expression of p-ERK1/2 relative to t-ERK1/2. The results were presented as the means ± standard deviations (*n* = 3; ^#^*P* < 0.05, ^##^*P* < 0.01 compared with the RA group). JPYS, Jian-Pi-Yi-Shen formula; ERK1/2, extracellular signal-regulated kinase 1/2; RA, renal anemia; LJPYS, low dose Jian-Pi-Yi-Shen (1.5 g/kg/d); HJPYS, high dose Jian-Pi-Yi-Shen (6.0 g/kg/d).

It has been reported that the Raf/MEK/ERK signaling pathway directly affects the synthesis of HIF-*α* protein such that it induces the expression of EPO gene [21]. We further investigated whether this regulatory pathway was involved in JPYS-treated rats. As presented in Figure 5, compared with the sham group, the phosphorylation of ERK1/2 protein in the RA group was downregulated, but the difference was not statistically significant. In administration of JPYS, the phosphorylation levels of ERK1 and ERK2 were significantly increased compared with the RA group.

### 3.4. Effects of JPYS on Iron Recycling

**Figure 6:**
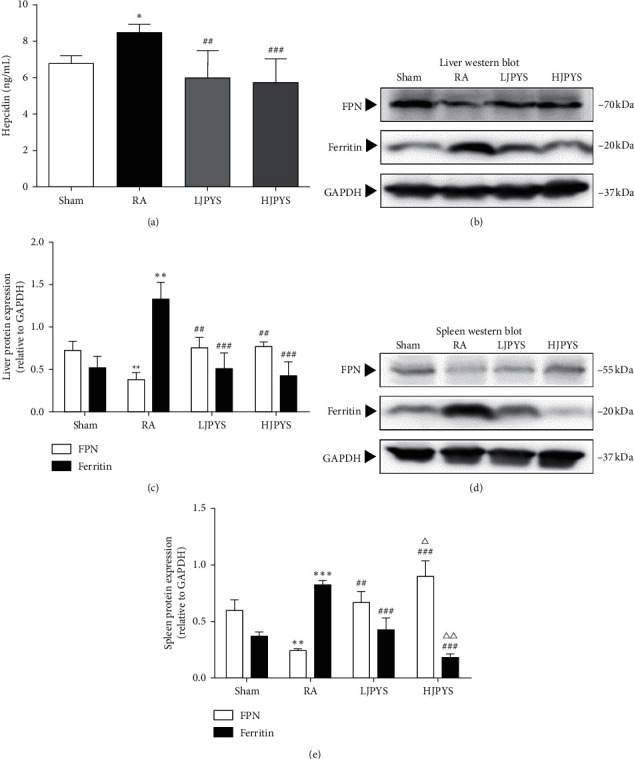
Effects of JPYS on the serum hepcidin level, and the proteins expression of FPN and ferritin in rats. (a) The level of hepcidin in serum from different groups (n = 6). (b) Representative Western blot images of FPN and ferritin in the liver. (c) Liver proteins expression of FPN and ferritin relative to GAPDH. (d) Representative Western blot images of FPN and ferritin in the spleen. (e) Spleen proteins expression of FPN and ferritin relative to GAPDH. The results were presented as the means ± standard deviations (*n* = 6 or 3; ^*∗*^*P* < 0.05, ^∗∗^*P* < 0.01, ^∗∗∗^*P* < 0.001 compared with the sham group; ^##^*P* < 0.01, ^###^*P* < 0.001 compared with the RA group; ^Δ^*P* < 0.05, ^ΔΔ^*P* < 0.01 compared with the LJPYS group). JPYS, Jian-Pi-Yi-Shen formula; FPN, ferroportin; GAPDH, glyceraldehyde-3-phosphate dehydrogenase; RA, renal anemia; LJPYS, low dose Jian-Pi-Yi-Shen (1.5 g/kg/d); HJPYS, high dose Jian-Pi-Yi-Shen (6.0 g/kg/d).

Hepcidin, ferroportin (FPN), and ferritin are important regulators for iron recycling. The serum hepcidin concentration in CKD rats was significantly higher than that of sham rats, while the hepcidin level of JPYS-treated groups was significantly lower than that of the RA group (Figure 6(a)). Compared with sham rats, the expression of FPN protein in the liver and spleen of CKD rats was significantly decreased, while the protein expression of ferritin in CKD rats was significantly increased. In comparison with that in CKD rats, treatment with JPYS at two concentrations significantly enhanced the FPN expression, and the ferritin expression was dramatically decreased by JPYS. In the spleen, the FPN expression was significantly elevated in the HJPYS group than in LJPYS, and the ferritin expression was significantly declined in the HJPYS group than in LJPYS (Figures 6(b)–6(e)).

## 4. Discussion

The global prevalence of CKD is approximately 13%, amongst which 50% of patients with stage 4 or 5 CKD have anemia complication [22]. Unlike anemia of iron deficiency, anemic patients with CKD suffer from various factors such as EPO resistance, functional iron deficiency, and chronic inflammation [23]. In consideration of the safety of recombinant EPO and iron treatment, some novel therapies such as HIF stabilizers, prolyl hydroxylase domain (PHD) inhibitors, and hepcidin antagonists have emerged in recent years [24–27]. All these treatments are targeting HIF signaling that is associated with EPO expression and iron metabolism. HIF-*α* can be hydroxylated by PHD enzymes and then recognized by the von Hippel-Lindau protein- (VHL-) E3 ubiquitin ligase complex for degradation through proteasome [6]. Therefore, inhibition of PHD enzyme activity is conducive to HIF-*α* stabilization. Hepcidin, a small molecular peptide produced by the liver, is a key regulator of iron metabolism that is stimulated by iron overload and inflammation [28]. Hepcidin is also regulated by HIF via EPO-induced erythropoiesis [29]. In this paper, the effect of JPYS on CKD anemia was studied based on both EPO expression and iron metabolism.

After 90 days of oral treatment, JPYS significantly improved renal function and blood routine levels in CKD rats. The body and kidney weight of males were heavier than those of females, indicating the males grow faster than the females. Anemia-related parameters (RGB, HGB, and HCT) between the males and the females did not show a significant difference. Interestingly, the level of UPRO in females was higher than that of males in both LJPYS and HJPYS groups yet not in a significant difference. Whether JPYS treatments are more effective at kidney function in males remains to be further clarified. According to the pathological examination, renal structural damage and interstitial fibrosis in CKD rats were significantly inhibited by JPYS treatment. The occurrence of renal interstitial fibrosis is accompanied by the decrease of fibroblasts, resulting in the reduction of EPO expression. In this study, JPYS significantly reduced the fibrotic area in CKD rats, the effect of which might involve the activation of HIF-2*α* in the kidney. In support of this notion, a recent study has shown that long-term activation of HIF-2*α* can inhibit the progression of renal fibrosis and improve renal function [30].

Of note, activating the HIF pathway promotes endogenous EPO production [17]. It has been found that there are three isoforms of HIF-*α*, among which HIF-2*α* is the key regulator of endogenous EPO gene transcription [12, 17]. In this study, detecting the changes in the expression of HIF-2*α* and EPO proteins in the kidney, we found that these protein levels in CKD rats had no significant differences compared with the sham group. The HIF-2*α* level in the RA group was even slightly higher than that in the sham group, while the EPO level in the RA group was slightly lower than that in the sham group. We assumed this might be resulted from the compensatory increase by residual kidney of rats. One clinical study showed that the EPO level in patients with CKD anemia was generally normal or slightly elevated [31], which was consistent with our experimental results. After finding that JPYS significantly increased the expression of HIF-2*α* and EPO proteins in the kidney, we also detected the HIF-2*α* and EPO proteins in the liver. When the function of the kidney is limited and it is unable to synthesize enough EPO, hepatic HIF-2*α* is assumed as the major role in regulating serum EPO levels [32]. Consistent with this, the expression of hepatic EPO protein of CKD rats was significantly low, and the level of HIF-2*α* protein was slightly lower than that of the sham group. Notably, after JPYS intervention, the expression of hepatic HIF-2*α* and EPO proteins was significantly increased.

Moreover, in association with HIF and its upstream regulatory cascade, one vitro experiment found that the Raf/MEK/ERK signaling pathway regulated the translation of HIF-*α*, while by blocking the expression of p-ERK1/2 protein, the transcription of EPO was inhibited [21]. In this study, it was found that the phosphorylation level of ERK1/2 in both RA and sham groups were low, while the phosphorylation level of ERK1/2 in the JPYS treatment groups was significantly increased. These results implicate that JPYS may promote the accumulation of HIF-2*α* protein by stimulating the phosphorylation of ERK1/2. In this experiment, we also detected the expressions of PHD1, PHD2, and PHD3 in the kidney and liver and found no significant differences in the protein expression among each group (Supplementary Figure 3). Therefore, we speculate that JPYS-induced HIF signaling pathway activation is not mediated by inhibiting the PHD pathway but by activating the Raf/MEK/ERK pathway.

Recent studies have found that hypoxia signals correlate erythropoiesis with iron homeostasis [33], which brings new insights into the treatment of CKD anemia. FPN is the only known substance exporting inorganic iron in mammalian cells, while excess iron can be stored in ferritin protein, and their expression is closely monitored under normal physiological conditions [34]. The binding of hepcidin to FPN induces the internalization and inactivation of FPN [35], which leads to the retention of iron in cells. Increased expression of ferritin protein and declined expression of FPN protein were observed in the liver and spleen of 5/6 nephrectomized rats, which indicated that excess iron in the spleen and liver was stored by ferritin and could not be released through FPN. These findings suggest that systemic iron recycling disorder is presented in CKD rats and further reduce iron utility for erythropoiesis. In this experiment, we found that JPYS significantly increased FPN protein expression and reduced ferritin protein expression, which indicated an improvement of utilization of iron stores in the liver and spleen. Besides, the overexpression of serum hepcidin reverted to its original level by JPYS treatment, ensuring the systemic and intracellular iron homeostasis. In line with this notion, previous studies showed that JPYS could suppress proinflammatory cytokines production in 5/6 nephrectomized rats [36]. The hepcidin expression in the liver can be induced by the upregulation of cytokines, most notably IL-6 and LPS [27], whereas the increased systemic hepcidin level can act on macrophages, preventing the release of iron recovered from senescent red blood cells into plasma [37]. This could also explain the expression of iron recycling proteins in macrophages of the liver and spleen of CKD rats. In addition, it has been reported that the expression of hepcidin, FPN, and ferritin in the liver is involved in HIF signaling [38–40]. Stabilized HIF can regulate increased FPN and decreased hepcidin and ferritin, which implies that the expression of hepcidin and iron recycling proteins are associated with the activation of JPYS-induced HIF-2*α* signaling. Increasing evidences show that acquiring the essential iron for erythropoiesis is mainly targeting HIF signaling management [33, 41, 42].

## 5. Conclusions

In summary, our data indicate that JPYS can correct CKD anemia through induction of EPO production and regulation of iron metabolic targets and the mechanism of which involves in the translational control of HIF-2*α* protein accumulation via ERK signaling. These findings provide evidence for the use of JPYS as a novel therapy for CKD anemia.

## Data Availability

The data used to support the findings of this study are available from the corresponding author upon request.

## Supplementary Materials

Supplementary Materials Supplementary Figure 1: HPLC-MS/MS chromatograms of the JPYS extract. The denotation peaks 1–10 were acteoside (1), liquiritin (2), rosmarinic acid (3), salvianolic acid A (4), calycosin (5), formononetin (6), rhein (7), dioscin (8), atractylenolide I (9), and tanshinone IIA (10). Representative chromatograms are shown, *n* = 6. Supplementary Figure 2: effects of JPYS on the serum Ca and P levels in rats (*n* = 6, ^*∗∗∗*^*P* < 0.001 compared with the sham group; ^#^*P* < 0.05, ^###^*P* < 0.001 compared with the RA group). Abbreviations: JPYS, Jian-Pi-Yi-Shen formula; Ca, calcium; P, phosphorus; RA, renal anemia; LJPYS, low dose Jian-Pi-Yi-Shen (1.5 g/kg/d); HJPYS, high dose Jian-Pi-Yi-Shen (6.0 g/kg/d). Supplementary Figure 3: effects of JPYS on the proteins expression of PHD1, PHD2, and PHD3 in rats. (a) Representative Western blot images of PHD1, PHD2, and PHD3 in kidneys. (b) Kidney proteins expression of PHD1, PHD2, and PHD3 relative to *β*-actin. (c) Representative Western blot images of PHD1, PHD2, and PHD3 in livers. (d) Liver proteins expression of PHD1, PHD2, and PHD3 relative to *β*-actin. The results are presented as the means ± standard deviations (*n* = 3). JPYS, Jian-Pi-Yi-Shen formula; PHD, prolyl hydroxylase domain; RA, renal anemia; LJPYS, low dose Jian-Pi-Yi-Shen (1.5 g/kg/d); HJPYS, high dose Jian-Pi-Yi-Shen (6.0 g/kg/d). .
