# Supplementary material for: Jian-Pi-Yi-Shen Regulates EPO and Iron Recycling Protein Expressions in Anemic Rats with Chronic Kidney Disease: Accumulation of Hypoxia Inducible Factor-2α via ERK Signaling
Source: Evid Based Complement Alternat Med. 2020 Oct 30;2020:8894257. doi: 10.1155/2020/8894257 (PMC7647762; doi:10.1155/2020/8894257)
Supplement: Supplementary Materials — Supplementary Figure 1: HPLC-MS/MS chromatograms of the JPYS extract. The denotation peaks 1–10 were acteoside (1), liquiritin (2), rosmarinic acid (3), salvianolic acid A (4), calycosin (5), formononetin (6), rhein (7), dioscin (8), atractylenolide I (9), and tanshinone IIA (10). Representative chromatograms are shown, n = 6. Supplementary Figure 2: effects of JPYS on the serum Ca and P levels in rats (n = 6, ∗∗∗P < 0.001 compared with the sham group; #P < 0.05, ###P < 0.001 compared with the RA group). Abbreviations: JPYS, Jian-Pi-Yi-Shen formula; Ca, calcium; P, phosphorus; RA, renal anemia; LJPYS, low dose Jian-Pi-Yi-Shen (1.5 g/kg/d); HJPYS, high dose Jian-Pi-Yi-Shen (6.0 g/kg/d). Supplementary Figure 3: effects of JPYS on the proteins expression of PHD1, PHD2, and PHD3 in rats. (a) Representative Western blot images of PHD1, PHD2, and PHD3 in kidneys. (b) Kidney proteins expression of PHD1, PHD2, and PHD3 relative to β-actin. (c) Representative Western blot images of PHD1, PHD2, and PHD3 in livers. (d) Liver proteins expression of PHD1, PHD2, and PHD3 relative to β-actin. The results are presented as the means ± standard deviations (n = 3). JPYS, Jian-Pi-Yi-Shen formula; PHD, prolyl hydroxylase domain; RA, renal anemia; LJPYS, low dose Jian-Pi-Yi-Shen (1.5 g/kg/d); HJPYS, high dose Jian-Pi-Yi-Shen (6.0 g/kg/d). . [file 8894257.f1.pdf]

Jian-Pi-Yi-Shen Regulates EPO and Iron Recycling Protein Expressions in Anemic Rats with Chronic Kidney Disease: Accumulation of Hypoxia Inducible Factor-2α via ERK Signaling

Fochang Wang [1,2#], Huimin Yu [3#], Shiying Huang [1], Lin Zheng [2], Ping Zheng [1,2], Shangbin Zhang [1,2], Shunmin Li [1*], Jianping Chen [1, 2*]

Affiliations:

[1] The Fourth Clinical Medical College of Guangzhou University of Chinese Medicine, Shenzhen 518033, China

[2] Shenzhen Key Laboratory of Hospital Chinese Medicine Preparation, Shenzhen Traditional Chinese Medicine Hospital, Shenzhen 518033, China

[3] School of Medicine, Shenzhen University, Shenzhen 518060, China

Fochang Wang and Huimin Yu are co-first authors.

***Correspondence should be addressed to** Jianping Chen; E-mail: lycjp@126.com and Shunmin Li; zyylishunmin@163.com

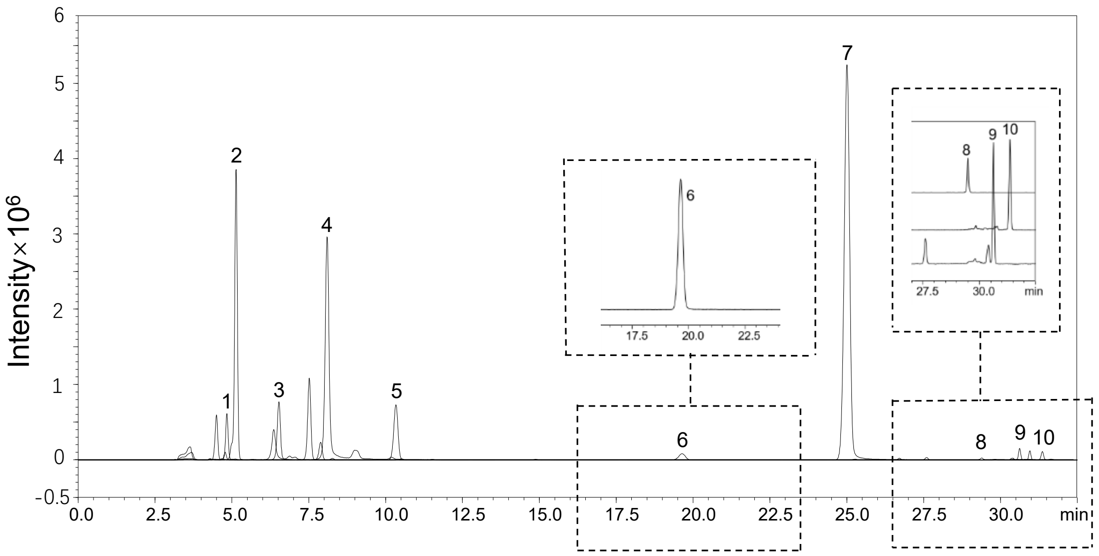

Fig S1: HPLC-MS/MS chromatograms of JPYS extract. The denotation peaks 1-10 were acteoside (**1**), liquiritin (**2**), rosmarinic acid (**3**), salvianolic acid A (**4**), calycosin (**5**), formononetin (**6**), rhein (**7**), dioscin (**8**), atractylenolide I (**9**), tanshinone IIA (**10**). Representative chromatograms are shown, *n* = 6.

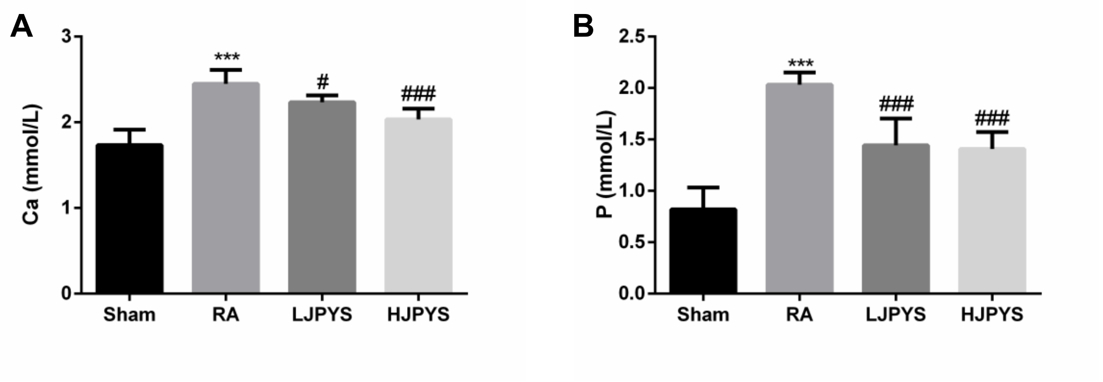

Fig S2: Effects of JPYS on the serum Ca and P levels in rats. $^{***}P < 0.001$ compared with the Sham group; $^{\#}P < 0.05$, $^{\#\#\#}P < 0.001$ compared with the RA group. Abbreviations: JPYS, Jian-Pi-Yi-Shen formula; Ca, calcium; P, phosphorus; RA, renal anemia; LJPYS, low dose Jian-Pi-Yi-Shen (1.5 g/kg/d); HJPYS, high dose Jian-Pi-Yi-Shen (6.0 g/kg/d).

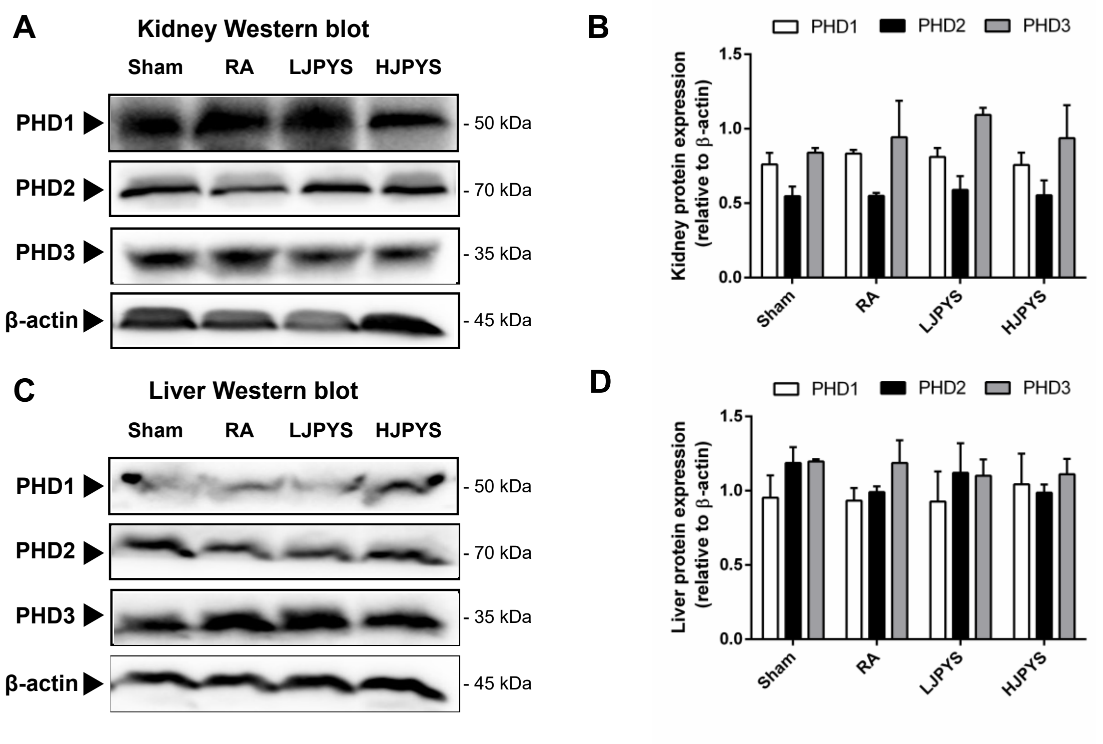

Fig S3: Effects of JPYS on the proteins expression of PHD1, PHD2 and PHD3 in rats. (A) Representative western blot images of PHD1, PHD2 and PHD3 in kidneys. (B) Kidney proteins expression of PHD1, PHD2 and PHD3 relative to β-actin. (C) Representative western blot images of PHD1, PHD2 and PHD3 in livers. (D) Liver proteins expression of PHD1, PHD2 and PHD3 relative to β-actin. The results were presented as the means ± standard deviations (n = 3). JPYS, Jian-Pi-Yi-Shen formula; PHD, prolyl hydroxylase domain; RA, renal anemia; LJPYS, low dose Jian-Pi-Yi-Shen (1.5 g/kg/d); HJPYS, high dose Jian-Pi-Yi-Shen (6.0 g/kg/d).
